# Supplementary material for: Indolent primary cutaneous B-cell lymphomas resemble persistent antigen reactions without signs of dedifferentiation
Source: Nat Commun. 2026 Feb 4;17:2366. doi: 10.1038/s41467-026-69210-9 (PMC12979821; doi:10.1038/s41467-026-69210-9)
Supplement: Supplementary file 1 — Supplementary Information [file 41467_2026_69210_MOESM1_ESM.pdf]

Indolent primary cutaneous B cell lymphoma represent persistent antigen reactions without signs of dedifferentiation - Supplementary Materials

Johannes Griss MD, PhD[1*], Sabina Gansberger MSc[1], Inigo Oyarzun[1], Martin Simon[1], Mathias C. Drach MD[1], Vy Nguyen MSc[1], Lisa E. Shaw MSc[1], Ulrike Mann[1], Stefanie Porkert MD[1], Matthias Farlik PhD[1], Wolfgang Weninger MD[1], Werner Dolak, MD[2], Bertram Aschenbrenner, PhD[1], Beate M. Lichtenberger, PhD[1], Shawn Ziegler-Santos[1], Christine Wagner[1], Ingrid Simonitsch-Klupp MD[3], Stephan N. Wagner MD[1], Constanze Jonak MD[1], Patrick M. Brunner MD, MSc[4*]

1 Department of Dermatology, Medical University of Vienna, Vienna, Austria

2 Division of Gastroenterology and Hepatology, Department of Internal Medicine 3, Medical University of Vienna, Austria

3 Department of Pathology, Medical University of Vienna, Austria

4 Department of Dermatology, Icahn School of Medicine at Mount Sinai, New York, USA

Table of Contents

Supplementary Figure 1 2
Supplementary Figure 2 3
Supplementary Figure 3 5
Supplementary Figure 4 6
Supplementary Figure 5 7
Supplementary Figure 6 8
Supplementary Figure 7 9
Supplementary Figure 8 10
Supplementary Tables 12

Supplementary Figure 1

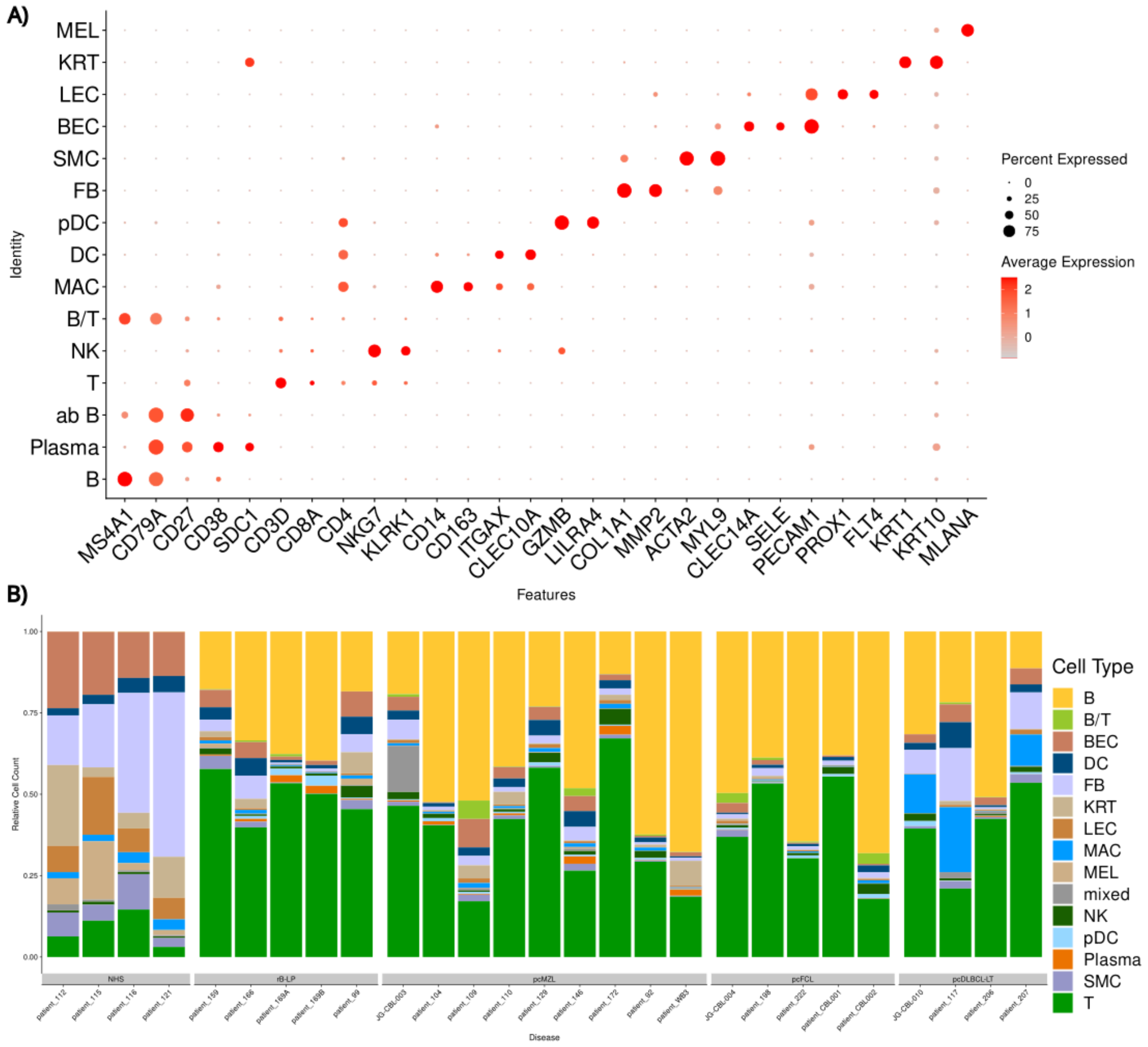

Analysis of the complete scRNA-seq data from the CBCL samples. A) Dot plots of cell clusters from the integration of all cutaneous samples (n=27) displaying average gene expression (red color) and frequency (circle size) of selected canonical cell type markers. **B)** Relative proportion of cell types in each individual cutaneous sample.

Supplementary Figure 2

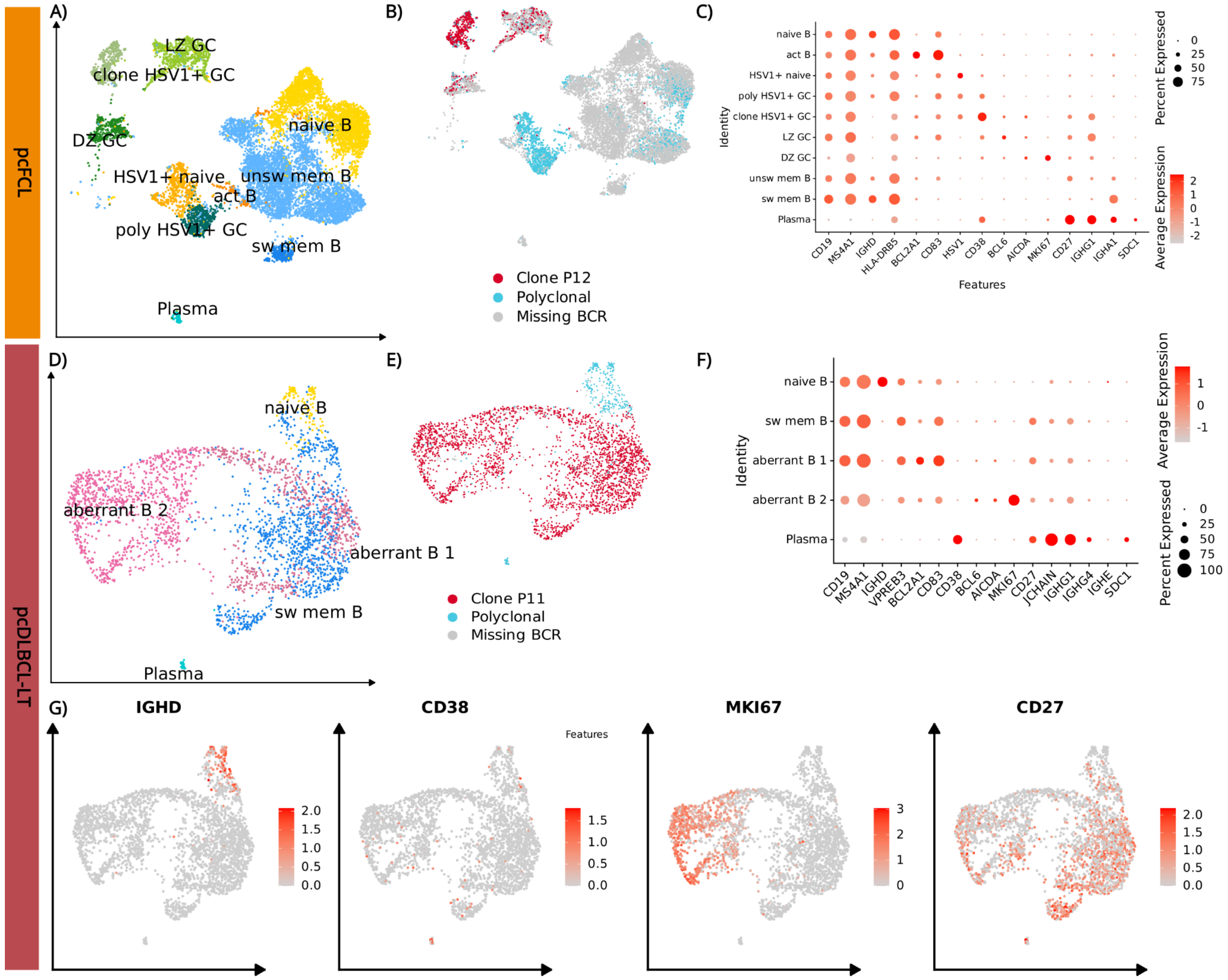

Reanalysis of extracted B cells from public data published by Ramelyte *et al.* consisting of one patient with pcFCL and one patient with pcDLCBL-LT. A) UMAP embedding of the pcFCL sample. **B)** Results of the BCR analysis for the pcFCL sample. Clonally expanded cells are highlighted as “Clone P12”. **C)** Dot plot representing the expression of key markers for cell type identification. **D)** UMAP embedding of the pcDLBCL-LT sample. **E)** Results of the BCR analysis. The top expanded clone is highlighted as “Clone P11”. **F)** Key markers used for cell type identification. **G)** Feature plot displaying the spatial expression of key markers, highlighting the aberrant phenotype of the majority of expanded B cells.

Supplementary Figure 3

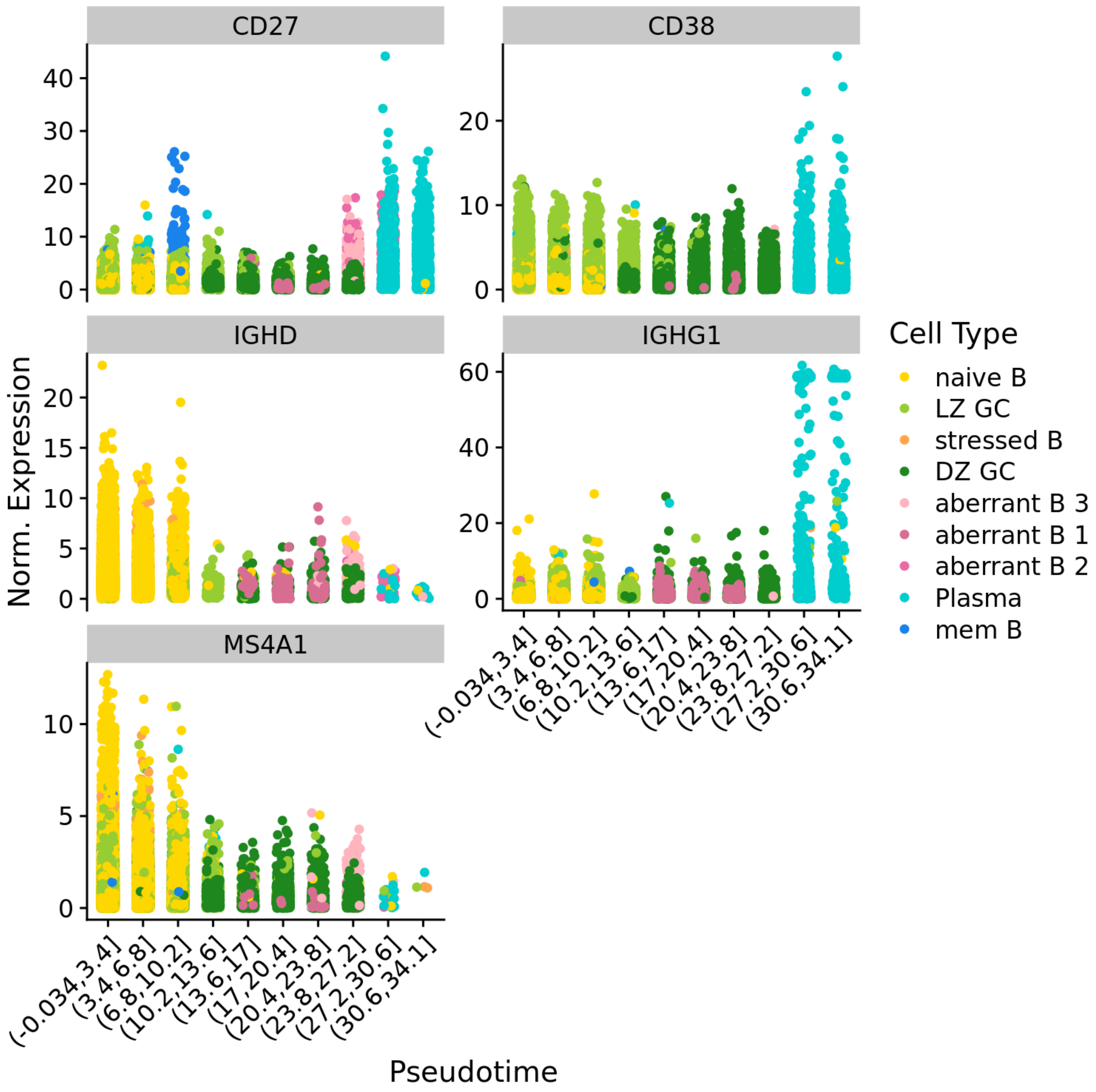

Expression values of key B cell development markers *vs.* the derived pseudotime from all cutaneous samples (n=23). Individual points represent individual cells. Colors represent the respective cell phenotypes.

Supplementary Figure 4

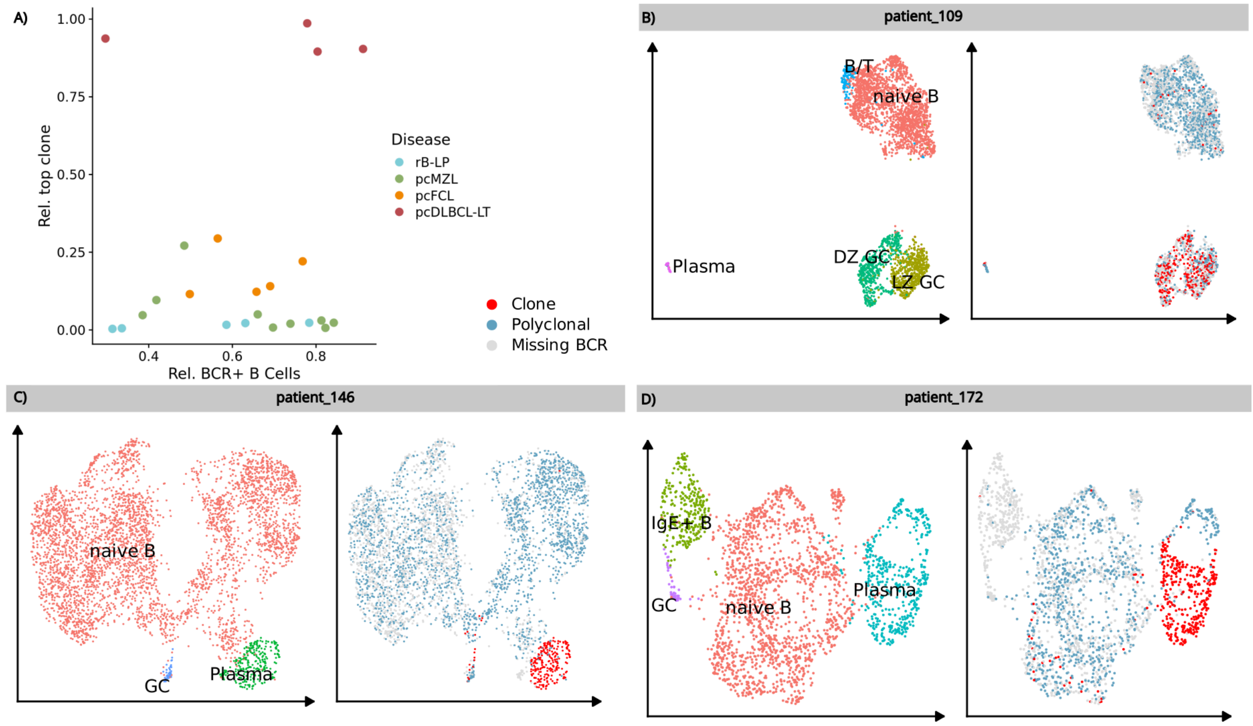

Analysis of outliers in the clonal expansion estimate in pcMZL samples. A) Proportion of B cells with BCR information *vs.* the relative proportion of the top clone of the B cell infiltrates with BCR information for each sample (n=23). Color represents the respective diseases. There were three pcMZL samples with a clonal expansion of 9%, 17% and 30%. Yet, these higher rates of clonally expanded B cells correlated with the lowest BCR capture of less than 50% of B cells in these samples. **B, C, D)** UMAP embedding of a subclustering of the B cells from samples 109, 146, and 172. In all samples, B cells without BCR information were found in the naive B cell clusters, indicating that these mainly contained polyclonal B cells. Colors represent the cell annotation in the left panel, and the BCR information in the right one.

Supplementary Figure 5

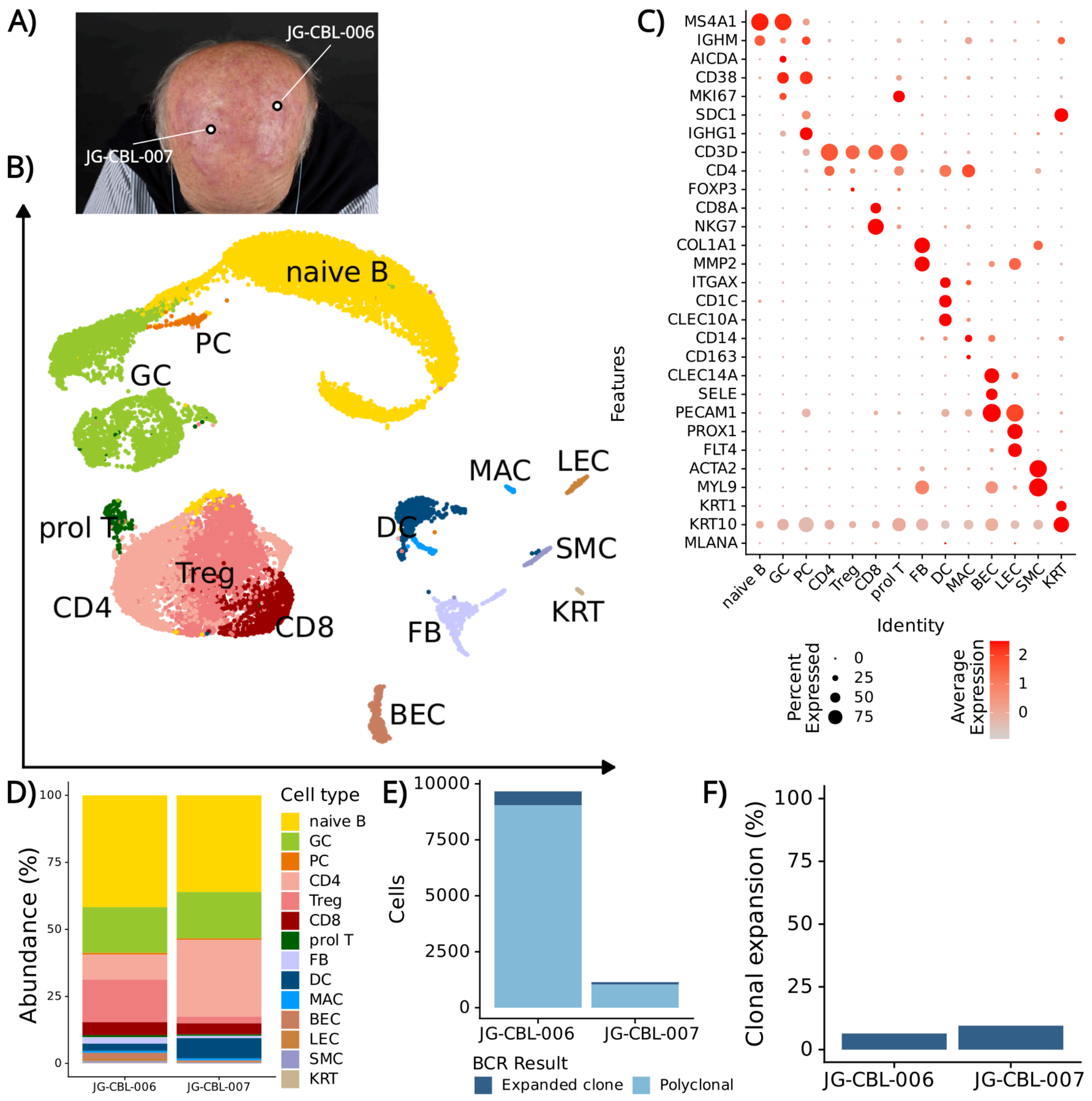

Evaluation of spatial heterogeneity within rB-LP lesions. A) Sites of the two biopsies taken simultaneously. **B)** UMAP embedding showing the cell type annotation based on **C)** canonical markers. **D)** The relative abundance of B cell phenotypes was comparable between both samples. **E)** Total number of recovered B cells differed considerably between both biopsies. **F)** Despite different numbers of recovered B cells, the estimated clonal expansion was comparable between both sites (6% for JG-CBL-006 and 9% for JG-CBL-007).

Supplementary Figure 6

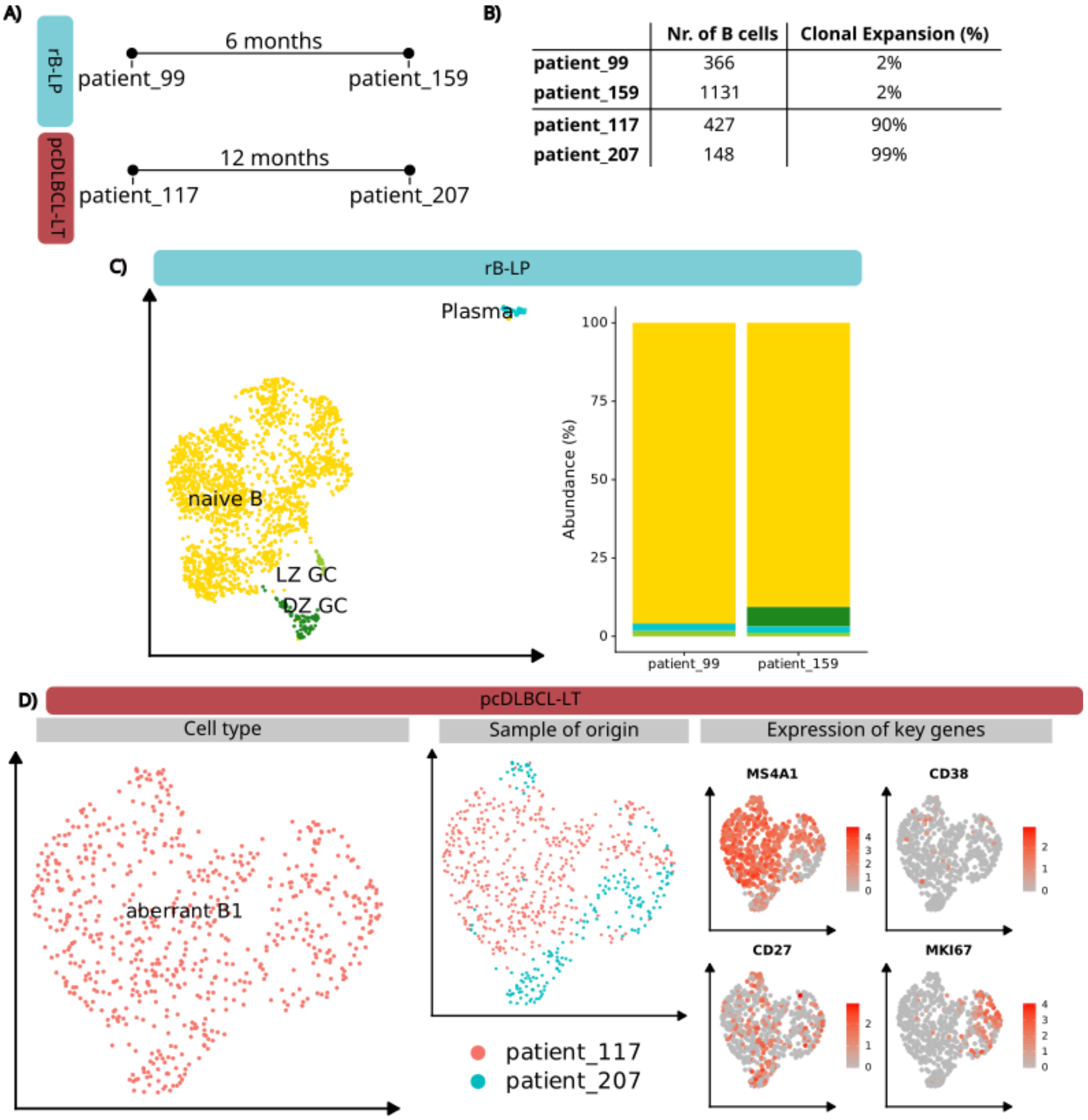

	Nr. of B cells	Clonal Expansion (%)
**patient_99**	366	2%
**patient_159**	1131	2%
**patient_117**	427	90%
**patient_207**	148	99%

Assessment of the change of clonal expansion through time. A) We acquired consecutive samples within 6 and 12 months from two patients with rB-LP and pcDLBCL-LT respectively. **B)** Number of B cells and the estimate clonal expansion based on the BCR data for each sample. **C)** UMAP embedding and phenotypic distribution of matched rB-LP samples highlighting the similarity of samples. **D)** UMAP embedding of pcDLBCL-LT samples which only contained aberrant B cells. Expression of key markers indicating the aberrant B cell phenotype.

Supplementary Figure 7

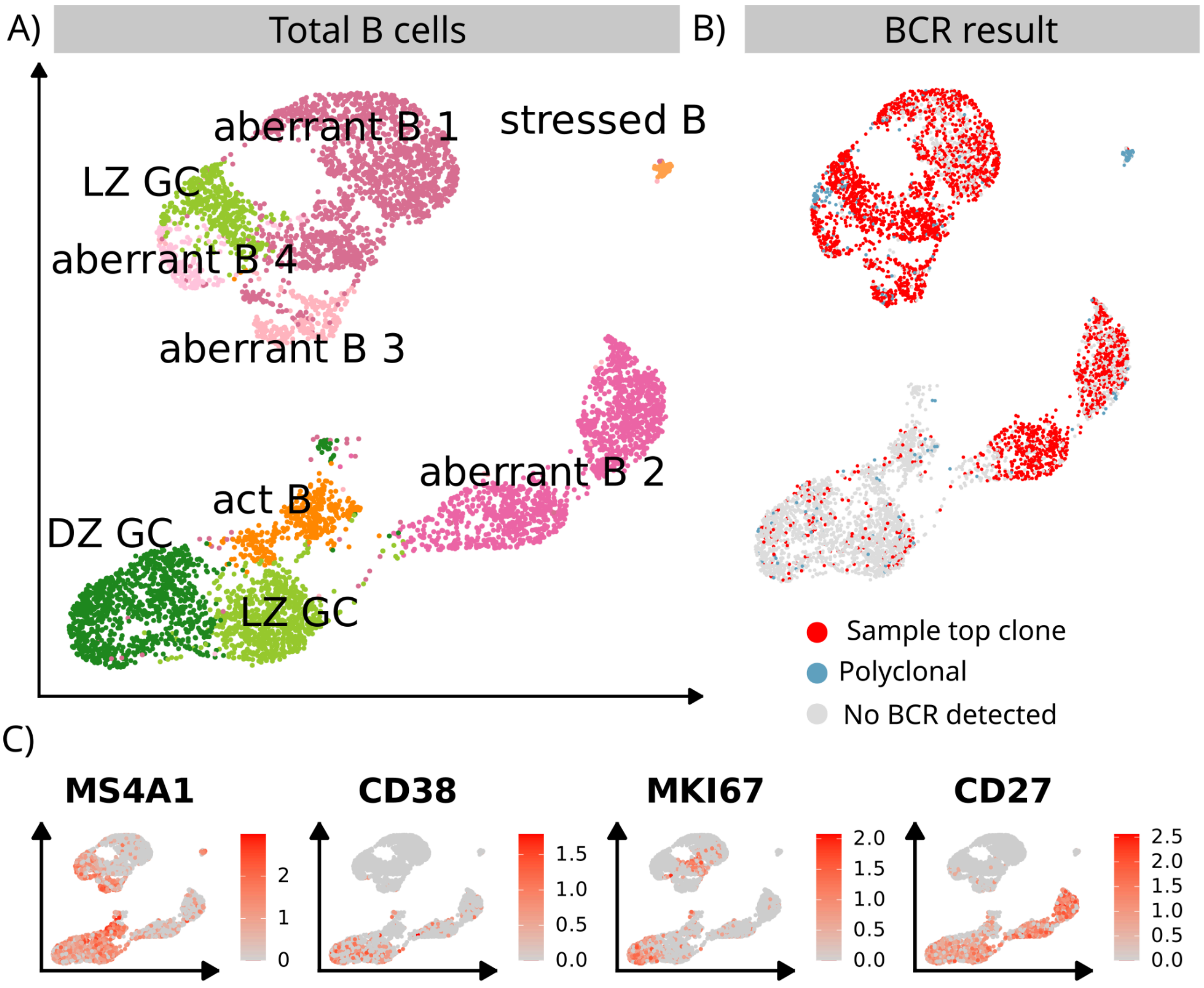

Separate analysis of all B cells from the pcDLCBL-LT samples. A) UMAP embedding of the subclustering of the B cells from the pcDLBCL-LT samples. **B)** Results of the BCR sequencing. **C)** Expression of key B cell markers that are not compatible with canonical B cell subtypes.

Supplementary Figure 8

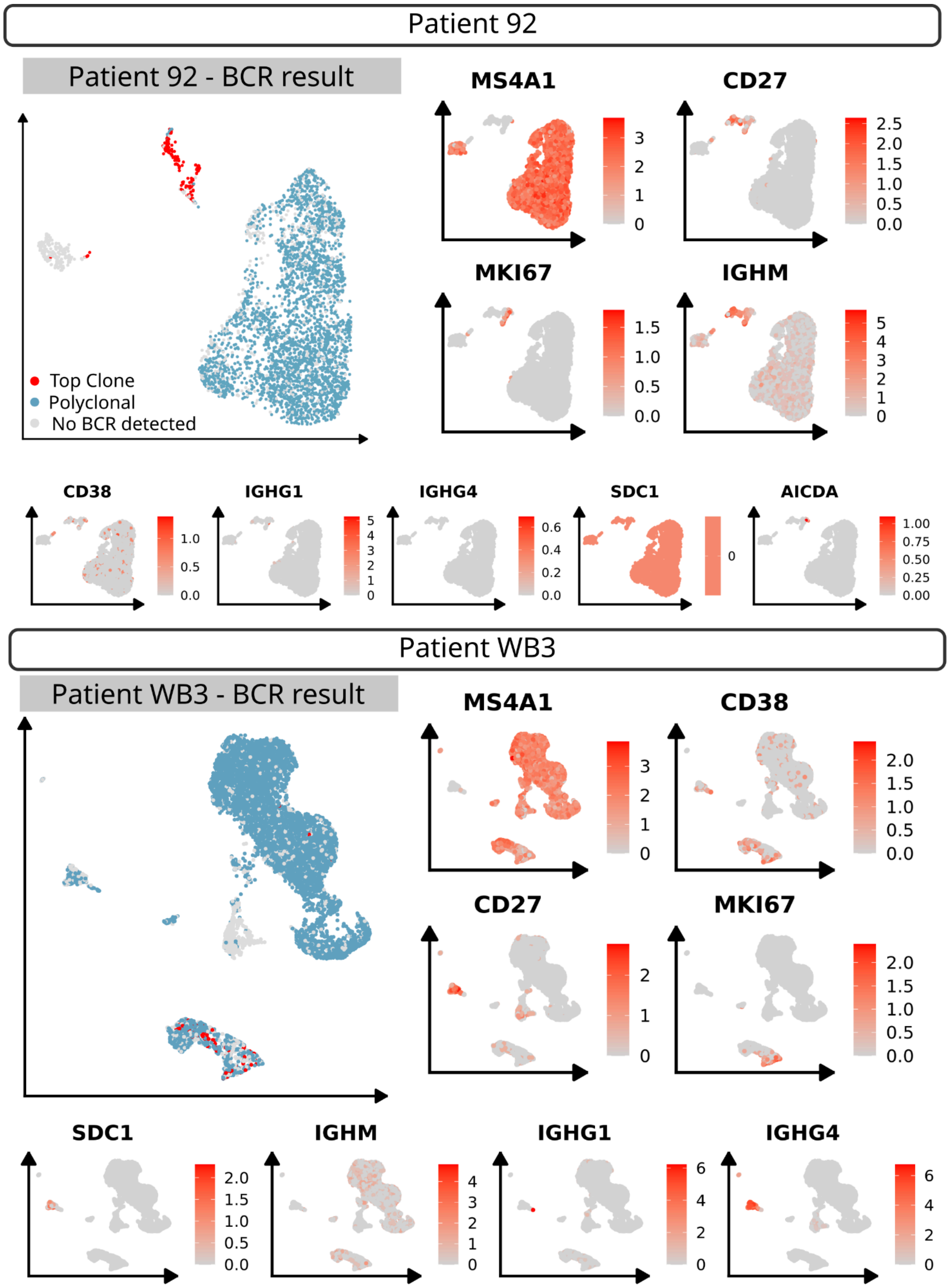

Subclustering of B cells from IgM+ pcMZL samples. Top panel) Results from patient 92. Right panel shows the results of the BCR analysis with expression of key B cell markers on the right. **Lower panel)** Results from patient WB3. Results from BCR sequencing on the right with expression of key B cell markers.

Supplementary Tables

Supplementary Table 1 Patient baseline characteristics at time of sampling.

Subject ID	Age	Sex	Race	Histopathological diagnosis	Location	Disease duration (years)	Ongoing treatment	Previous treatments	Disease stage
112	51	F	White	Healthy control	Trunk	n.a.	n.a.	n.a.	n.a.
115	48	M	White	Healthy control	Trunk	n.a.	n.a.	n.a.	n.a.
116	56	F	White	Healthy control	Trunk	n.a.	n.a.	n.a.	n.a.
121	44	F	White	Healthy control	Trunk	n.a.	n.a.	n.a.	n.a.
92	72	M	White	pcMZL	Upper arm	8 years	None	Rituximab	T1bN0M0
104	33	M	White	pcMZL	Trunk	3 years	None	Clarithromycin	T1bN0M0
110	47	M	White	pcMZL	Trunk	13 years	None	Rituximab	T3aN0M0
129	38	M	White	pcMZL	Shoulder	14 years	None	Clarithromycin, surgery	T3aN0M0
146	47	M	White	pcMZL	Upper arm	1 year	None	Surgery	T1aN0M0
172	72	W	White	pcMZL	Lower thigh	2 years	None	Surgery, radiotherapy	T1bN0M0
WB3	60	M	White	pcMZL	Neck	1 year	None	None	T1bN0M0
109	79	F	White	pcMZL	Back / Shoulder	1 year	None	Radiotherapy	T2aN0M0
JG-CBL-003	79	M	White	pcMZL	Back	3 years	None	Surgery	T1bN0M0
99 + 159	30	M	White	rB-LP	Upper arms	1 year	None	None	n.a.
166	47	F	White	rB-LP	Face	16 years	None	Glucocorticosteroids, hydroxychloroquine, apremilast	n.a.
169A + 169B	56	M	White	rB-LP	Upper leg	1 year	None	Surgery	n.a.
198	77	M	White	pcFCL	Scalp	1 year	None	None	T2aN0M0
222	32	M	White	pcFCL	Face	< 1 year	None	Surgery	T2aN0M0
JG_CBL_001	51	M	White	pcFCL	Scalp	3 years	None	None	T2cN0M0
JG_CBL_002	72	W	White	pcFCL	Face	< 1 year	None	None	T2cN0M0
JG-CBL-004	43	M	White	pcFCL	Scalp	10 years	None	Rituximab	T2cN0M0
206	74	M	White	pcDLBCL-LT	Lower leg	<1 year	None	None	T2bN0M0
117 + 207	78	F	White	pcDLBCL-LT	Forearm and lower leg	<1 year	None	Radiotherapy, R-CHOP	T3aN0M0
JG-CBL-010	79	F	White	pcDLBCL-LT	Lower leg	<1 year	None	None	T2bN0M0
200	62	M	White	Gastric MALT	--	< 1 year	None	HP-Eradication	n.a.
201	31	M	White	Gastric MALT	--	1 year	None	HP-Eradication	n.a.
210	59	F	White	Gastric MALT	--	2 years	None	HP-Eradication	n.a.
211	63	F	White	Gastric MALT	--	1 year	None	HP-Eradication	n.a.
