# Supplementary material for: Indolent primary cutaneous B-cell lymphomas resemble persistent antigen reactions without signs of dedifferentiation
Source: Nat Commun. 2026 Feb 4;17:2366. doi: 10.1038/s41467-026-69210-9 (PMC12979821; doi:10.1038/s41467-026-69210-9)
Supplement: Supplementary file 2 — Description of Additional Supplementary Files [file 41467_2026_69210_MOESM2_ESM.pdf]

Description of Additional Supplementary Files:

Supplementary Data 1: Marker genes for each annotated cell type as reported by the FindMarkers function from the CBCL sample integration.

Supplementary Data 2: CDR3 sequences for each clone per sample.

Supplementary Data 3: Marker genes for each annotated B cell type as reported by the FindMarkers function from the complete B cell integration across all entities.

Supplementary Data 4: Differentially expressed genes between the top clones of the different entities.

Supplementary Data 5: Complete results of the CellChat interaction analysis.
