# Supplementary material for: Indolent primary cutaneous B-cell lymphomas resemble persistent antigen reactions without signs of dedifferentiation
Source: Nat Commun. 2026 Feb 4;17:2366. doi: 10.1038/s41467-026-69210-9 (PMC12979821; doi:10.1038/s41467-026-69210-9)
Supplement: Supplementary file 8 — Reporting Summary [file 41467_2026_69210_MOESM8_ESM.pdf]

nature portfolio

Corresponding author(s): Griss J, Brunner PM

Last updated by author(s): Dec 28, 2025

Reporting Summary

Nature Portfolio wishes to improve the reproducibility of the work that we publish. This form provides structure for consistency and transparency in reporting. For further information on Nature Portfolio policies, see our Editorial Policies and the Editorial Policy Checklist.

Statistics

For all statistical analyses, confirm that the following items are present in the figure legend, table legend, main text, or Methods section.

n/a	Confirmed	
☐	☒	The exact sample size (*n*) for each experimental group/condition, given as a discrete number and unit of measurement
☐	☒	A statement on whether measurements were taken from distinct samples or whether the same sample was measured repeatedly
☐	☒	The statistical test(s) used AND whether they are one- or two-sided<br>*Only common tests should be described solely by name; describe more complex techniques in the Methods section.*
☐	☒	A description of all covariates tested
☐	☒	A description of any assumptions or corrections, such as tests of normality and adjustment for multiple comparisons
☒	☐	A full description of the statistical parameters including central tendency (e.g. means) or other basic estimates (e.g. regression coefficient) AND variation (e.g. standard deviation) or associated estimates of uncertainty (e.g. confidence intervals)
☒	☐	For null hypothesis testing, the test statistic (e.g. *F*, *t*, *r*) with confidence intervals, effect sizes, degrees of freedom and *P* value noted<br>*Give P values as exact values whenever suitable.*
☒	☐	For Bayesian analysis, information on the choice of priors and Markov chain Monte Carlo settings
☒	☐	For hierarchical and complex designs, identification of the appropriate level for tests and full reporting of outcomes
☒	☐	Estimates of effect sizes (e.g. Cohen's *d*, Pearson's *r*), indicating how they were calculated

Our web collection on statistics for biologists contains articles on many of the points above.

Software and code

Policy information about availability of computer code

Data collection	Data was generated from patient samples recruited at the Department of Dermatology, Medical University of Vienna, additionally, the following public datasets were processed as well: EGAS00001006052, EGAS00001004904, GSE182436
Data analysis	Raw data from scRNA-seq was preprocessed using Cell Ranger version 7.0.1, multiplex immunohistochemistry data was initially processed using InForm 2.4, Perkin Elmer, all subsequent data analyses were performed using R version 4.1.0 and the following R libraries to process the scRNA-seq data: Seurat (v 5.0.1), Biostrings (v 2.68), DecontX, scDlbFinder

For manuscripts utilizing custom algorithms or software that are central to the research but not yet described in published literature, software must be made available to editors and reviewers. We strongly encourage code deposition in a community repository (e.g. GitHub). See the Nature Portfolio guidelines for submitting code & software for further information.

Data

Policy information about availability of data

All manuscripts must include a data availability statement. This statement should provide the following information, where applicable:

- Accession codes, unique identifiers, or web links for publicly available datasets
- A description of any restrictions on data availability
- For clinical datasets or third party data, please ensure that the statement adheres to our policy

The scRNA-seq data generated in this study have been deposited in the GEO database under accession code GSE218861 (https://www.ncbi.nlm.nih.gov/geo/query/acc.cgi?acc=GSE218861). Data from healthy control samples are available on GEO under GSE173205 (https://www.ncbi.nlm.nih.gov/geo/query/acc.cgi?

acc=GSE173205)80. The raw scRNA-seq data are protected and are not available due to data privacy laws. The CBCL scRNA-seq data used in this study are available in the European Genome-Phenome Archive (EGA) database under accession code EGAD00001006829 (https://ega-archive.org/datasets/EGAD00001006829). The sFCL scRNA-seq data used in this study are available in the EGA database under accession code EGAS00001006052 (https://ega-archive.org/datasets/EGAS00001006052). The sDLBCL scRNA-seq data used in this study are available in the GEO database under accession code GSE182436 (https://www.ncbi.nlm.nih.gov/geo/query/acc.cgi?acc=GSE182436). Source data are provided with this paper.

Research involving human participants, their data, or biological material

Policy information about studies with human participants or human data. See also policy information about sex, gender (identity/presentation), and sexual orientation and race, ethnicity and racism.

Reporting on sex and gender	No sex or gender based analyses were performed. Due to the rarity of the investigated diseaes, all patients with the respective diagnoses willing to participate in this study were included.
Reporting on race, ethnicity, or other socially relevant groupings	Due to the demographics in Austria, all patients were white caucasian. Race or ethnicity were not part of the eligibility criteria.
Population characteristics	Detailed characteristics for each study participant are present in supplementary table 1
Recruitment	Patients were recruited in the cutaneous lymphoma outpatient clinic at the Department of Dermatology of the Medical University of Vienna. All patients with the respective diagnoses willing to participate in this study were included.
Ethics oversight	The study protocol was approved by the Ethics Committee of the Medical University of Vienna (EK 1360/2018)

Note that full information on the approval of the study protocol must also be provided in the manuscript.

Field-specific reporting

Please select the one below that is the best fit for your research. If you are not sure, read the appropriate sections before making your selection.

[x] Life sciences   [ ] Behavioural & social sciences   [ ] Ecological, evolutionary & environmental sciences

For a reference copy of the document with all sections, see nature.com/documents/nr-reporting-summary-flat.pdf

Life sciences study design

All studies must disclose on these points even when the disclosure is negative.

Sample size	Due to the rarity of the diseases, all patients willing to participate in this study were included. No dedicated sample size calculation was performed.
Data exclusions	No samples were excluded from the analyses.
Replication	All key findings presented in the manuscript could be replicated across all investigated samples.
Randomization	No randomization was performed.
Blinding	Immunohistochemistry data was analysed in a blinded fashion.

Reporting for specific materials, systems and methods

We require information from authors about some types of materials, experimental systems and methods used in many studies. Here, indicate whether each material, system or method listed is relevant to your study. If you are not sure if a list item applies to your research, read the appropriate section before selecting a response.

Materials & experimental systems

n/a	Involved in the study	
[ ]	[x]	Antibodies
[x]	[ ]	Eukaryotic cell lines
[x]	[ ]	Palaeontology and archaeology
[x]	[ ]	Animals and other organisms
[x]	[ ]	Clinical data
[x]	[ ]	Dual use research of concern
[x]	[ ]	Plants

Methods

n/a	Involved in the study	
[x]	[ ]	ChIP-seq
[x]	[ ]	Flow cytometry
[x]	[ ]	MRI-based neuroimaging

Antibodies

Antibodies used

CD19 BV711 (clone SJ25C1), CD20 AF700 (clone 2H7), CD24 PE-CF594 (clone ML5), CD27 BV421 (clone M-T271), CD38 APC (clone HIT2), CD138 PE (clone MI15), IgD PE-Cy7 (clone IA6-2), IgG FITC (clone G18-145), IgM BV605 (clone G20-127) (all BD biosciences)

Validation

Respective stainings without primary antibodies were used as a negative control. Along with tissue arrays, serial sections of melanoma specimens and normal controls were stained to assess reproducibility. At equal Ab-concentrations, TSA-based visualization is expected to yield a higher number of positive cells as compared to conventional immunofluorescence. We therefore established TSA-based visualization of primary Abs on control tonsil tissue, the golden standard for lymphocyte antigen detection in pathology, and performed a comparison for each Ab to validated staining patterns in human tonsil (as to the Human Protein Atlas, available from www.proteinatlas.org). Thereafter, we balanced the signal through dilution of the primary Abs to obtain staining levels and cell frequencies comparable to conventional immunofluorescence staining. The dilution of the CD19 antibody was optimized to allowing for the detection of CD19low plasma cell-like cells as compared to patterns and frequencies obtained by CD138+ pooled IgA/IgG+ stainings on human tonsil and melanoma. In multiplex stainings, single primary Ab stainings were run in parallel to control for false positive results through incomplete Ab-TSA complex-"stripping" and false negative results through antigen masking (by incubation with multiple primary Abs, "umbrella-effect"). Spillover effects were controlled for anti-CD20-Ab stainings on tonsil with different Opal fluorophores by signal detection in adjacent components/channels and thereafter for exposure time settings upon acquisition of multiplex-stained tissue sections.

Plants

Seed stocks

Report on the source of all seed stocks or other plant material used. If applicable, state the seed stock centre and catalogue number. If plant specimens were collected from the field, describe the collection location, date and sampling procedures.

Novel plant genotypes

Describe the methods by which all novel plant genotypes were produced. This includes those generated by transgenic approaches, gene editing, chemical/radiation-based mutagenesis and hybridization. For transgenic lines, describe the transformation method, the number of independent lines analyzed and the generation upon which experiments were performed. For gene-edited lines, describe the editor used, the endogenous sequence targeted for editing, the targeting guide RNA sequence (if applicable) and how the editor was applied.

Authentication

Describe any authentication procedures for each seed stock used or novel genotype generated. Describe any experiments used to assess the effect of a mutation and, where applicable, how potential secondary effects (e.g. second site T-DNA insertions, mosiacism, off-target gene editing) were examined.
